# High Expressed Emotion and Warmth among Families of Patients with Schizophrenia in Greece

**DOI:** 10.3390/healthcare10101957

**Published:** 2022-10-07

**Authors:** Georgios Avraam, Maria Samakouri, Anthimos Tzikos, Aikaterini Arvaniti

**Affiliations:** 1 Department of Psychiatry, Medical School, Democritus University of Thrace, 681 00 Alexandroupolis, Greece; 2 Department of Psychiatry, University General Hospital of Alexandroupolis, 681 00 Alexandroupolis, Greece

**Keywords:** schizophrenia, family, expressed emotion, warmth

## Abstract

Expressed emotion (EE) is an established prognostic factor for relapse in schizophrenia. Through critical comments (CC), hostility (H) and emotional overinvolvement (EOI), a relative can be rated as high or low EE, but the role of warmth should also be evaluated in order to consider the influence of a positive affect within the family context. In this study, EE was assessed in a sample of 48 relatives of patients with schizophrenia using the Camberwell Family Interview (CFI). Questionnaires assessing coping (brief-COPE), their wellbeing (World Health Organization Well-Being Index WHO-5) and the socio-demographic variables were also administered. Relatives who expressed a higher level of warmth were found to make fewer CC (5.2 ± 4.6 vs. 8.4 ± 4.6, *p* = 0.009) and have, on average, higher EOI scores (3.2 ± 1.0 vs. 1.9 ± 1.1, *p* = 0.002) than those who expressed no or very little warmth. High EE was found to be associated with having fewer family members (*p* = 0.035), while relatives with a higher level of education expressed less warmth (*p* = 0.007). Relatives with a low level of warmth had higher maladaptive coping scores and tended to score worse for their overall wellbeing in comparison to relatives who showed a higher level of warmth (28.4 ± 5.0 vs. 24.1 ± 5.2, *p* = 0.006 and 39.1 ± 20.4 vs. 51.3 ± 22.0, *p* = 0.073, respectively). Since the role of warmth is important, it should be taken into account when designing family interventions, independently from lowering EE. Customized interventions to promote warmth and the routine screening of relatives are recommended.

## 1. Introduction

Expressed emotion (EE) is a scientific construct that allows the evaluation of the family environment, particularly that of patients with schizophrenia. The gold standard for assessing EE is the Camberwell Family Interview (CFI), which evaluates the critical comments (CC), hostility (H), emotional overinvolvement (EOI), positive remarks and warmth that the relative expresses towards the patient. The term “high expressed emotion (high EE)” is used to describe families whose members express themselves towards patients with a high level of criticism (CC), emotional overinvolvement (EOI) or hostility (H) [1]. Patients with schizophrenia who live in a “high EE” family environment have a higher rate of relapse. This rate is almost double for patients who return, after discharge, to a high (EE) family in comparison to those who return to a low EE environment, as demonstrated in a meta-analysis of 26 studies [2]. In the majority of EE studies, the patient’s variables, such as their gender or the severity and type of their psychopathology, do not seem to be associated with the EE (low or high) [3,4,5,6]. These findings, concerning the patients with schizophrenia, do not seem to be in line with findings for patients suffering from other mental disorders and their relatives, as it is described in the study of Giannouli et al. in a Greek sample of patients with mild Alzheimer’s disease and their caregivers, where the caregivers’ and patients’ characteristics were associated. The results highlighted that the caregivers were biased against the estimated financial capacity of women patients [7].

The negative aspects of EE, especially those relating to a high level of CC, have been thoroughly examined and their value, integrity and robustness in predicting symptoms relapse have been proven [8,9]. Although the CFI offers information about both positive and negative features of the family environment, the focus has been primarily on its negative aspects because of their predictive role in relapse in schizophrenia. In the initial studies conducted in this area, a high level of warmth was found to be associated with high EOI [10], and this association probably discouraged researchers from including warmth in their studies for many years. This has, to some extent, made the families of patients with schizophrenia be viewed as either a negative or neutral background for the patient [11]. On the other hand, the role of relatives who act as caregivers for people with mental illness, and especially for patients with schizophrenia, can be positive and protective. The contribution made by carers in the United Kingdom (UK) is thought to have an economic value of 132 billion GBP per year, and almost one out of four of these caregivers support a person with mental health problems [12]. In a 14-year follow-up study conducted in rural China, it was found that patients with schizophrenia who have a caregiver have a lower percentage of homelessness and a higher percentage of survival than those who do not [13]. Although there are indications that high levels of warmth, even in families with high EE, are associated with lower admission rates [14], only in the recent literature has emphasis been placed on warmth and its protective role in the course of schizophrenia. Lee at al. found better clinical outcomes at 6- and 12-month follow-ups after a first psychotic episode in cases where warmth and positive affect were higher, and they highlighted the protective role and positive affect of warmth in families that already have a warm emotional environment and stronger ties [15]. Warmth is a neglected protective family factor [16]. Family interventions have kept their main focus on the EE construct and do not take into account the needs and emotional responses of relatives [17]. The family caregivers for patients with schizophrenia report higher stress levels in comparison with family caregivers for relatives with other mental illnesses [18]. A higher perceived burden and a lower quality of life are common amongst caregivers for patients with schizophrenia [19]. Moreover, the coping of the caregivers for individuals with schizophrenia appears to be associated with numerous variables, including EE [20]. Coping relates to the way individuals detect, appraise, deal with, learn from and respond to stressful situations. If the relative has more adjustable ways of managing the patient’s mental disorder, he/she will be less likely to be critical towards the patient [21]. The need to include warmth in family interventions has motivated the research team to explore it in relation to schizophrenia. Based on the connection between EE and distress [22] and the relationship between warmth and positive outcomes in psychosis [23], the aim of the present study is to assess the rate of EE and warmth in the relatives of patients with schizophrenia in Greece and to explore the relationship between warmth and the other aspects of EE along with wellbeing, coping and sociodemographic characteristics.

## 2. Materials and Methods

### 2.1. Sample

The sample of this study consists of 48 key family members who are caregivers of patients with schizophrenia, according to the International Classification of Diseases, 10th Revision (ICD-10), which is the official and exclusive classification system in the Greek health care system, including all hospital and mental health services. The patients and their families were living in the capital city of Athens and in the Evros area, in Northern Greece. These areas represent the urban and semirural and rural parts of Greece. The patients and their relatives were users of psychiatric services at either Evaggelismos Hospital in Athens or at the University General Hospital in Alexandroupolis, in the Evros area. The research team approached the relatives that accompanied the patient to the outpatient department during their visit. The relatives were then briefed about the study and if they agreed to participate, they were interviewed by the trained interviewer and completed its forms. The relatives had to be 18–70 years of age, have fluency in Greek and meet the criteria for being a key relative. Key relatives of the patient are considered the ones that have lived in the same household with the patient at least for the last six months and have daily contact with him/her. The selection process occurred in a prefixed working day of the month, initially in Evaggelismos Hospital and afterwards in the University General Hospital of Alexandroupolis, and a maximum of the first two consenting relatives a day could be recruited. The patient’s inclusion criteria were: (1) the indicated diagnosis. A diagnosis was confirmed by the treating psychiatrist who was the responsible physician for each participant and by the psychiatric outpatient department of each hospital, who had the responsibility to establish the diagnosis. (2) A stable diagnosis for a six-month period at least. Only patients who were visiting the outpatient department of the two aforementioned hospitals for at least six months and whose diagnosis of schizophrenia remained unchanged after their initial visit to the mental health services were included in the study. The exclusion criteria, regarding the patients, were considered to be the following: (1) a severe somatic illness that could interfere with the psychiatric diagnosis, (2) any degree of mental retardation and (3) a dual diagnosis of schizophrenia along with substance use or a history of substance use.

### 2.2. Questionnaires

The following questionnaires were administered:

1. A socio-demographic characteristics form collecting relatives’ gender, age, education, marital status, religion, employment status, income, current residence, financial status, family structure, relation to the patient and professional support (psychiatrist, psychologist, group therapy, etc.).
2. Camberwell Family Interview (CFI): EE is assessed using the CFI [24], as used in previous Greek studies [25]. The CFI is the gold standard for assessing EE and is a semi-structured interview of the patient’s key relatives. The CFI has five subscales:
  - Criticism: the number of critical comments that the relative makes about the patient.
  - Hostility: a global scale that shows the relative’s generalized critical attitude and dislike towards the patient as a person and/or shows the relative’s rejection of the patient (0 = absence of hostility, 1 = hostility as a generalization, 2 = hostility as rejection and 3 = both generalization and rejection).
  - Emotional overinvolvement: a global rating taken from the whole interview and scored from 0 to 5. This shows how intrusive, self-sacrificing and/or emotionally over-reactive the relative is towards the patient.
  - Positive remarks: a frequency count of the number of positive comments that the relative makes in the interview.
  - Warmth: a global scale for the warmth the relatives express towards the patient. The aspects considered are the tone of voice, spontaneity, sympathy, concern, empathy and interest in the person. Warmth is scored on a scale ranging from 0 to 5 (0 = no warmth, 1 = very little warmth, 2 = some warmth, 3 = moderate warmth, 4 = moderately high warmth and 5 = high warmth). For the scope of this data analysis, answers were unified in two categories: some or higher warmth = 1 (scores 2, 3, 4, and 5) and no/very little warmth = 0 (scores 0, 1).

The relatives were scored as having a high EE according to the criteria that are described in the CFI [24]: those who made six or more critical comments during the interview and/or expressed hostility (hostility score: 1, 2 or 3) and/or had a rating of 3 or more in the EOI scale were rated as having a high EE.

Family members were interviewed with the CFI by the first author and their responses were audio-taped and turned into a transcript. All the audiotapes and the transcripts were later reviewed and scored by the first author according to the CFI coding. The rater was trained in London in the CFI coding by Christine Vaughn and had a training reliability criterion of 0.9 in all EE scales.

3. Brief COPE: COPE and its different versions are the most widely used scales for assessing coping [26]. The Greek version of the Brief COPE, which has been shown to have adequate psychometric properties, was used [27]. The Brief COPE is a 28-item scale, and the answers to each item are given on a four-point Likert-type scale, ranging from 1 (I have not been doing this at all) to 4 (I have been doing this a lot). The 28 items represent 14 2-item subscales [28,29]. The higher the score on a certain subscale, the higher the use of the specific coping type for the specific stress and problem. The coping styles can be expected to be adaptive or maladaptive and the creator of the questionnaire, Carver C.S., invited researchers to adapt the items of the COPE according to the researcher’s hypotheses, samples and situations [28]. In the current study, eight COPE scales were considered adaptive (namely acceptance, humor, active coping, positive reframing, planning, use of instrumental support, use of emotional support and religion) and six maladaptive (namely behavioral disengagement, self-distraction, self-blame, denial, venting and substance use) [30].
4. The World Health Organization Well-Being Index (WHO-5): this is one of the most popular questionnaires used to assess wellbeing. It has been translated into over 30 languages and has been implemented in five continents. It is a useful tool for assessing subjective wellbeing, taking into account the last fourteen days, and consists of 5 simple questions that are answered on a 6-point Likert-type scale: all of the time = 5, most of the time = 4, more than half of the time = 3, less than half the time = 2, some of the time = 1 and at no time = 0. After scoring the above five questions, there will be a total score of 0–25. This first score is multiplied by the number 4 and a final score of 0–100 is reached. Lower scores represent a worse wellbeing and higher scores represent a better wellbeing [31]. Moreover, the WHO-5 Well-Being Index is used as a screening tool for depression [32].

### 2.3. Statistical Analysis

A descriptive analysis of the samples was conducted, using means ± SDs for continuous variables and percentages for categorical variables. Due to the small sample size, non-parametric statistics were used to examine the association among the variables and Cohen’s D and phi as a measurement of the effect size. Fisher’s exact test was performed to examine the association between the categorical demographic characteristics and the bivariate version of warmth (no or very little warmth vs. some or higher warmth) and the levels of EE (low vs. high). The Mann–Whitney test was used to examine the mean difference of the continuous demographic variables, WHO-5 and Brief COPE between the levels of warmth and the EE of the relatives. The statistical significance level was set at 5% or 10% when it was combined with a medium effect size, which was also measured in order to evaluate the significance of the relations [33,34]. The IBM SPSS Statistics 24 software was used for the analysis.

## 3. Results

### 3.1. Description of the Sample

The majority of the sample of relatives (48) were women (Ν = 32, 66.67%). The average age was 50.94 years (±13.89), with an average duration of education of 11.79 (±3.64) years. Most of the relatives (N = 43, 89.58%) were Christians and the rest were Muslims (N = 5, 10.42%). Out of the 32 female relatives, 26 were mothers, 5 were sisters and 1 was the grandmother of the patient, while of the 16 male relatives, 12 were fathers, 1 was a husband, 2 were full brothers and 1 was the stepbrother of the patient. Regarding their marital status, 27 (56.25%) of the relatives were married, 8 (16.67%) were single, 6 (12.50%) were divorced and 7 (14.58%) were widows/widowers. In total, 10 relatives (20.83%) had a monthly income of less than 500 euros, 24 (50%) had a monthly income ranging from 500 to 1000 euros, 8 (16.67%) had a monthly income between 1000 and 1500 euros and 6 (12.50%) had a monthly income of more than 1500 euros. Only seven (14.8%) had received any kind of support from an expert (psychiatrist, psychologist, group therapy, etc.) in the past.

### 3.2. Expressed Emotion

**Figure 1:**
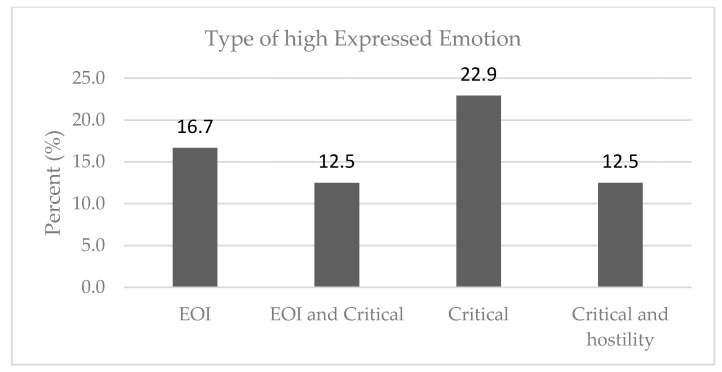
Percentage of each type of high expressed emotion (EOI = emotional overinvolvement).

As shown in Table 1, the majority of the sample of the relatives (64.6%) were found to have a high EE towards the patients. The percentages of the relatives who expressed each type of high EE are given in Figure 1, where it can be seen that 16.7% of the relatives expressed EOI, 12.5% were EOI and critical, 22.9% were critical and 12.5% were critical and hostile. Table 1 presents the relatives’ type of EE based on their demographic characteristics. Females tended to express a higher EE (71.9%) than the males (50.0%); however, the difference was not found to be statistically significant (*p* = 0.135) and the effect size was found to be between a small and medium effect (phi = 0.22). Mothers and fathers were found to express statistically significant levels of higher EE (74.4%) than brothers and sisters (22.2%, *p* = 0.006). Relatives that never received professional support, express a higher EE (87.5%) than those with previous or current professional support (60.0%). Even though the association was not found to be significant (*p* = 0.230), the effect size shows a small to medium effect (phi = 0.21). The number of household members was related with the level of EE, since relatives with a high EE came from families with fewer household members than those with a low EE (2.2 ± 0.9 vs. 2.8 ± 0.7, *p* = 0.035).

The mean values on the scales of the WHO-5 and Brief COPE based on the levels of the relatives’ EE are presented in Table 2. Relatives with a low EE had, on average, higher levels of wellbeing (56.0 ± 24.6) than those with a high EE (43.2 ± 19.4, *p* = 0,062). The differences were not found to be statistically significant at the level of 5%, but were at the level of 10%, which can be considered as an important relation since the corresponding Cohen’s D value (0.60) indicates more than a medium effect size. The mean values of maladaptive coping do not differ in a way which is statistically significant among the relatives with a low EE (24.4 ± 6.0) and a high EE (25.9 ± 5.1, *p* = 0.346); however, Cohen’s D value (0.28) indicates that there is more than a small effect.

### 3.3. Warmth

Table 3 presents the demographic characteristics of the relatives by the level of warmth they express. In total, 14 out of the 48 relatives (29.2%) expressed no or very little warmth, while the remaining 34 (70.8%) expressed at least some warmth (including some, moderate, moderately high and high warmth). Apart from education, none of the demographic characteristics were found to be significantly related to the relative’s level of warmth towards the patient. Relatives with higher levels of education expressed lower levels of warmth towards the patients (*p* = 0.007). Women tended to be more likely to express a higher level of warmth towards the patient (78.1% expressed at least some warmth) compared to men (56.3%), and relatives that received professional support were more likely to express at least some warmth (75.0%) than relatives without any experience of professional support (50.0%). The associations were not found to be statistically significant (*p* = 0.178 and *p* = 0.208); however, the corresponding phi measures indicate small to medium effect sizes (phi = 0.23 and phi = 0.21).

According to Table 4, there was a statistically significant relationship between the number of CC and the level of EOI and the levels of warmth. Relatives that showed no or very little warmth expressed, on average, more CC (8.4 ± 4.6) than those who expressed higher levels of warmth (5.2 ± 4.6, *p* = 0.009). Moreover, relatives who expressed higher levels of warmth had, on average, higher levels of EOI (3.2 ± 1.0) than those who showed no or very little warmth (1.9 ± 1.1, *p* = 0.002). Relatives who express some or higher levels of warmth give, on average, more positive remarks (1.2 ± 1.6) than the relatives who express no or very little warmth (0.4 ± 0.7, *p* = 0.179), which was not found to differ significantly. However, the corresponding Cohen’s D value was found to be 0.57, indicating a medium effect size, and thus we suggest that warmth and positive remarks are related substantially. The level of EE and hostility was not found to be related with the levels of warmth. Relatives with a low EE showed some or a higher level of warmth (82.4%), whereas 64.5% of those with a high EE showed some or a higher level of warmth. Relatives with no hostility expressed higher levels of warmth (76.3%) than relatives with hostility (50.0%). The differences were not found to be statistically significant (*p* = 0.320 and 0.130); however, the effect size measurements show small to medium associations (phi = 0.19 and phi = 0.24).

Relatives with higher levels of warmth (some warmth or higher levels of warmth) tended to have, on average, a higher level of wellbeing than that of relatives who expressed no or very little warmth (51.3 ± 22.0 vs. 39.1 ± 20.4, *p* = 0.073). The mean difference was found to be statistically significant at the level of 10%; however, the corresponding Cohen’s D value (0.56) indicates a medium effect size (Table 5). Relatives with higher scores in maladaptive coping strategies tended to express significantly lower levels of warmth. Relatives who expressed no or very little warmth had, on average, higher maladaptive coping scores than relatives who showed at least some warmth towards the patients (28.4 ± 5.0 vs. 24.1 ± 5.2, *p* = 0.006). No statistically significant association was found between the adaptive coping strategies used and the level of warmth.

## 4. Discussion

More than half of our sample expressed a high level of EE towards the patients, although the majority of relatives expressed at least some warmth towards them. Relatives who showed higher levels of warmth had, on average, higher levels of EOI and lower scores for maladaptive coping strategies than those who expressed lower levels of warmth. Caregivers who showed lower levels of warmth expressed higher levels of CC. The relative’s level of education seemed to be the only demographic characteristic that was significantly related (negatively) with their level of warmth towards the patients.

The predictive value of high EE in the patient’s psychotic relapse has been established across cultures universally and the differences among different cultures are mainly in the conceptualization of the EE, since there is no normative EE experience. The caregiving is in great part culturally determined, making comparisons among cultures difficult [35]. There are differences in Western countries, since the relatives are more critical there [22,36], but there are also differences in South and Northern Europe. In the Mediterranean region, there are a few studies that could provide an overview of the EE of the relatives of patients with schizophrenia and the range of high EE that appears to be mainly of the high EOI subcategory in relatives [14,37]. Similar studies conducted using the CFI and carried out in Mediterranean–South European countries, such as Italy, Spain and Serbia, found that high EE relatives ranged from 38% to 72% [14,38,39,40,41]. Mavreas et al. assessed the EE of 121 relatives of chronic patients with schizophrenia with the CFI in Greece and found that 70/121 (58%) had a low EE and 51/121 (42%) had a high EE [25].

Relatives that never received professional support tend to express a higher EE than those with previous or current professional support. Previous studies confirm the EE decrease of family members after psychoeducation and family interventions. Little knowledge and insufficient information regarding the mental disorder are considered to lead family members to express negative emotions [42,43].

In the present study, it was found that critical relatives showed less warmth towards the patients and that those with higher levels of EOI were more likely to express higher levels of warmth. It has been recorded, from the early CFI studies on EE, that high levels of warmth are associated with the co-occurrence of high EOI and low CC [10,44,45]. Moreover, high levels of warmth, even in families with a high EE, have been found to be associated with lower admission rates and have also been thought to be a protective factor against relapse in the context of a moderate EOI [14,46].

Our results indicate that warmth and positive remarks tend to be positively related. Positive remarks, just like warmth, were excluded from the determinant EE scales [10] and were neglected in the EE studies. Although positive remarks are not predictive of relapse [23], they can predict life satisfaction along with warmth [47]. Moreover, positive remarks are the only EE component that were found to be associated with less suicidal ideation [48] and are also associated with problem-solving skills and the constructive behavior of the relatives in the family environment [49,50]. Warmth is associated with EOI and positive remarks, and these three elements are characterized as positive communication [51]. O Brien et al. found that EOI, positive remarks and warmth are associated with a reduction in symptoms and an increase in social functioning in a three-month period in adolescents at risk of psychosis and argues that EOI could be developmentally appropriate as a family support in people who are at risk of psychosis in adolescence [52].

Females were found more likely to have a high EE than males and to express a higher level of warmth. This finding, although not statistically significant, is in line with previous results showing that women more often have a high EE [53] and, in particular, express higher levels of EOI [54], but at the same time they are more likely to express positive emotions than males and express them more easily [55]. Most of the study’s female participants were mothers (25/32). Mothers usually have higher levels of EOI than other relatives [56], but their warmth is associated with a higher life satisfaction and is a predictor of the life satisfaction of patients with schizophrenia [47].

Parents and other relatives from households with fewer members were found to express a statistically significant higher EE than siblings and relatives who live in large families. Koutra et al. found that one-parent families (predominantly a female parent) had higher levels of CC and family burden, arguing that a single parent has to finance, make decisions, stress and cope with the patient’s illness alone [54]. Although siblings do not identify themselves as caregivers [57], they seem to play an important role in the family, especially in the first psychotic episode of the patient, at which point they are usually teenagers or young adults [58]. Consequently, it has been argued that all family members should be included in family interventions in order to reduce the stress of the caregiver and also to manage stressful thoughts around future care [59].

Caregivers with low levels of warmth tend to perceive their wellbeing as lower, which is expressed by their score on the WHO-5 questionnaire. Considering that scoring low on the WHO-5 might indicate that the participant has depression, this result points towards mental suffering being associated with a low level of warmth and making it harder for the relative to have a positive attitude towards the patient. The mental health condition of the relative is probably important not only to them having a negative critical attitude but also in the positive effect that the warmth has. In the literature, depression in the relatives of patients has been associated with a high burden of care [60,61].

Caregivers that have a higher score for maladaptive coping show less warmth towards their relative with schizophrenia. It could be hypothesized that a caregiver with maladaptive coping mechanisms may find it more difficult to show warmth towards the patient when their overall experience of caregiving is negative. This could be in agreement with the findings of Doval et al. who reported that the adaptive coping strategies of the relative are correlated with a positive caregiving experience, while maladaptive coping strategies are associated with a negative experience, suggesting that better coping helps relatives to improve and appraise their experience better [62].

According to our results, relatives with a higher educational level appeared to express less warmth towards the patient. Although a higher level of education has been found to be related to better management and a lower caregiving burden [63], it could be the case that better education blocks the individual from expressing warmth. To the best of our knowledge, there are no previous studies confirming this result. Further research is needed in order to evaluate and replicate this finding.

The findings of our study highlight the need for interventions that aim to increase relatives’ warmth, such as improving their mental health and enhancing their coping skills. Bellack et al. found that patients with schizophrenia were not able to cope with the negativity of their relative, as expressed through criticism, while on the other hand they were able to perceive positive emotional displays from their relatives [64]. Therefore, it can be concluded that even though achieving a reduction in the criticism of all relatives is very important in order for interventions to succeed, the simultaneous promotion of warmth in all relatives is also of great value, since it can be beneficial independently of criticism. The emphasis on the negative factors and not the protective role of warmth could be disempowering for families [16].

Our study has some limitations, such as the lack of a structured interview to verify the diagnosis of schizophrenia. Neuroimaging, genetics, a neuropsychology assessment, personality and intelligence evaluation, electroencephalography, a drug - urine test to exclude organic or toxic psychosis were not included in the present study, as it is the case in many other EE studies to date [3,14,43,54], in which the diagnosis of schizophrenia has already been given by the treating physician, before the relatives, who are in the focus of the research interest, are recruited into the study. Moreover, in the present study and in order to reduce the possibility of including the relatives of individuals who did not have schizophrenia, relatives either of patients who used substances or had a shorter than 6-month follow up from the outpatient department were excluded from the sample.

Another limitation is the small sample size which did not allow us to perform a multivariate regression analysis of the data. On the other hand, the CFI studies have usually small sample sizes, possibly because the interview is too long. Additionally, cross-sectional studies such as the present one do not allow the identification of causal relations between the variables that are studied, they only highlight associations. Moreover, the fact that the EE of the relatives was not associated with patient’s characteristics is another limitation of this study. In fact, in the majority of EE studies, patients’ variables, such as their gender or the severity and type of their psychopathology, do not seem to be associated with the type of EE (low or high) of their close relatives [3,4,5,6]. Still, this is the first Greek study which focused especially on warmth and its value, even if high CC and/or high EOI exist in families at the same time.

## 5. Conclusions

Relatives who express a low level of warmth tend to make more CC, score worse in terms of wellbeing and adopt more maladaptive coping strategies to cope with the difficulties of being a caregiver for a person with schizophrenia. Family interventions should be designed to focus not only on lowering the EE of family members, but also on maintaining or enhancing their levels of warmth. Mental health services should be made easily accessible to caregivers and should take action to relieve their distress. High educational levels could block relatives from expressing warmth, but this finding needs to be replicated. The foresight shown by the creators of the CFI in including warmth in the ratings of EE, although it did not initially seem important, has given families the opportunity to participate actively and highlight the protective factors that are associated with the caregivers of patients with schizophrenia.

## Figures and Tables

**Table 1:** Demographic characteristics of the relatives based on their level of expressed emotion (EE) towards the patient (N = 48).

Demographic Characteristics		Low EE	High EE		
		N (%)	N (%)	*p*	Effect Size phi
EE		17 (35.4)	31 (64.6)		
Gender	Male	8 (50.0)	8 (50.0)	0.135	0.22
	Female	9 (28.1)	23 (71.9)		
Relation	Parent	10 (25.6)	29 (74.4)	**0.006**	0.43
	Sibling	7 (77.8)	2 (22.2)		
Marital status	Married	9 (33.3)	18 (66.7)	0.732	0.05
	Other	8 (38.1)	13 (61.9)		
Income	<1000	12 (35.3)	22 (64.7)	1.000	0.01
	>1000	5 (35.7)	9 (64.3)		
Profession	With their own income	13 (34.2)	25 (65.8)	0.704	0.08
	Without their own income (students, housekeepers, unemployed)	4 (44.4)	5 (55.6)		
Professional support	No	1 (12.5)	7 (87.5)	0.230	0.21
	Yes	16 (40.0)	24 (60.0)		
		**Mean ± Std. dev.**	**Mean ± Std. dev.**	***p***	**Effect Size** **Cohen’s D**
Age (years)		49.5 ± 17.4	51.7 ± 11.8	0.940	0.16
Education (years)		11.7 ± 4.3	11.9 ± 3.3	0.841	0.06
Number of household members		2.8 ± 0.7	2.2 ± 0.9	**0.035**	0.65

**Table 2:** World Health Organization Well-Being Index (WHO-5) and Brief COPE based on the relatives’ level of expressed emotion (EE) (N = 48).

	Low EE	High EE		
Scales	Mean ± Std. dev.	Mean ± Std. dev.	*P*	Effect Size Cohen’s D
WHO-5	56.0 ± 24.6	43.2 ± 19.4	**0.062**	0.60
Maladaptive coping	24.4 ± 6.0	25.9 ± 5.1	0.346	0.28
Adaptive coping	40.3 ± 6.9	41.5 ± 8.5	0.503	0.14

**Table 3:** Demographic characteristics of the relatives based on the level of warmth towards the patient (N = 48).

Demographic Characteristics		No or Very Little Warmth	Some or Higher Warmth		
		N (%)	N (%)	*p*	Effect Size phi
Warmth		14 (29.2)	34 (70.8)		
Gender	Male	7 (43.8)	9 (56.3)	0.178	0.23
	Female	7 (21.9)	25 (78.1)		
Relation	Parent	12 (30.8)	27 (69.2)	0.611	0.07
	Sibling	2 (22.2)	7 (77.8)		
Marital status	Married	8 (29.6)	19 (70.4)	0.936	0.01
	Other	6 (28.6)	15 (71.4)		
Income	<1000	9 (26.5)	25 (73.5)	0.728	0.09
	>1000	5 (35.7)	9 (64.3)		
Profession	With their own income	12 (31.6)	26 (68.4)	0.704	0.08
	(students, housekeepers, unemployed)	2 (22.2)	7 (77.8)		
Professional support	No	4 (50.0)	4 (50.0)	0.208	0.21
	Yes	10 (25.0)	30 (75.0)		
		**Mean ± Std. dev.**	**Mean ± Std. dev.**	***P***	**Effect Size** **Cohen’s D**
Age (years)		50.0 ± 13.3	51.3 ± 14.3	0.768	0.09
Education (years)		14.1 ± 1.9	10.9 ± 3.8	**0.007**	0.96
Number of household members		2.5 ± 1.1	2.4 ± 89.0	0.729	0.09

**Table 4:** Expressed emotion (EE) based on the level of relatives’ warmth.

		No or Very Little Warmth	Some or Higher Warmth		
		N (%)	N (%)	*p*	Effect Size phi
EE	Low	3 (17.6)	14 (82.4)	0.320	0.19
	High	11 (35.5)	20 (64.5)		
Hostility (2 categories)	No	9 (23.7)	29 (76.3)	0.130	0.24
	Yes (as generalization, rejection, or both)	5 (50.0)	5 (50.0)		
		**Mean ± Std. dev.**	**Mean ±Std. dev.**	***p***	**Effect Size** **Cohen’s D**
CC ^1^		8.4 ± 4.6	5.2 ± 4.6	**0.009**	0.70
EOI ^2^		1.9 ± 1.1	3.2 ± 1.0	**0.002**	1.18
Positive Remarks		0.4 ± 0.7	1.2 ± 1.6	0.179	0.57

^1^ Critical comments, ^2^ emotional overinvolvement.

**Table 5:** World Health Organization Well-Being Index (WHO-5) and Brief COPE based on the level of relatives’ warmth (N = 48).

	No or Very Little Warmth	Some or Higher Warmth		
Scales	Mean ± Std. dev.	Mean ± Std. dev.	*p*	Effect Size Cohen’s D
WHO-5	39.1 ± 20.4	51.3 ± 22.0	0.073	0.56
Maladaptive coping	28.4 ± 5.0	24.1 ± 5.2	**0.006**	0.83
Adaptive coping	42.1 ± 9.7	40.6 ± 7.2	0.544	0.19

## Data Availability

Not applicable.
